# Strontium Isotope Signals in Cremated Petrous Portions as Indicator for Childhood Origin

**DOI:** 10.1371/journal.pone.0101603

**Published:** 2014-07-10

**Authors:** Lise Harvig, Karin Margarita Frei, T. Douglas Price, Niels Lynnerup

**Affiliations:** 1 Laboratory of Biological Anthropology, Department of Forensic Medicine, Faculty of Health Sciences, University of Copenhagen, Copenhagen, Denmark; 2 National Museum of Denmark, Conservation and Natural Sciences Department, and The Danish National Research Foundation’s Centre for Textile Research (CTR) Prinsens Palæ, Copenhagen, Denmark; 3 Laboratory for Archaeological Chemistry, University of Wisconsin-Madison, Madison, Wisconsin, United States of America; University of Pennsylvania, United States of America

## Abstract

Dental enamel is currently of high informative value in studies concerning childhood origin and human mobility because the strontium isotope ratio in human dental enamel is indicative of geographical origin. However, many prehistoric burials involve cremation and although strontium retains its original biological isotopic composition, even when exposed to very high temperatures, intact dental enamel is rarely preserved in cremated or burned human remains. When preserved, fragments of dental enamel may be difficult to recognize and identify. Finding a substitute material for strontium isotope analysis of burned human remains, reflecting childhood values, is hence of high priority. This is the first study comparing strontium isotope ratios from cremated and non-cremated petrous portions with enamel as indicator for childhood origin. We show how strontium isotope ratios in the otic capsule of the petrous portion of the inner ear are highly correlated with strontium isotope ratios in dental enamel from the same individual, whether inhumed or cremated. This implies that strontium isotope ratios in the petrous bone, which practically always survives cremation, are indicative of childhood origin for human skeletal remains. Hence, the petrous bone is ideal as a substitute material for strontium isotope analysis of burned human remains.

## Introduction

Due to its high content of hydroxyapatite crystals [1] and absence of collagen, dental enamel has been of high informative value in studies concerning childhood origin and hence human mobility [2]–[7]. Harbeck et al. documented in a recent major study that Strontium (Sr) in cremated human bone retains its original biological isotopic composition, even when exposed to very high temperatures [8]. However, intact dental enamel is rarely preserved in cremated or burned human remains, due to the rapid destruction of the unprotected dental crowns during intense heat exposure [9]. When preserved, fragments of dental enamel may be difficult to recognize and identify as to a specific tooth or even of definite human origin. It is therefore of high priority to explore if there are other skeletal sources, that may survive burning, and which like teeth have a “locked-in” early signal.

**Figure 1:**
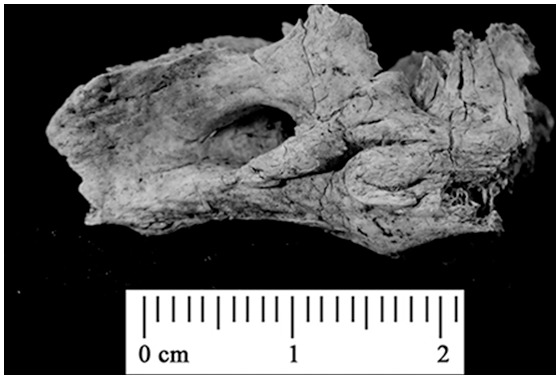
The petrous portion of the human temporal bone (*pars petrosa*) retains its morphology after cremation or similar intensive heat exposure, being one of the last bones of the body to burn. Here, a typical cremated petrous portion as found in cremated human remains is shown.

**Figure 2:**
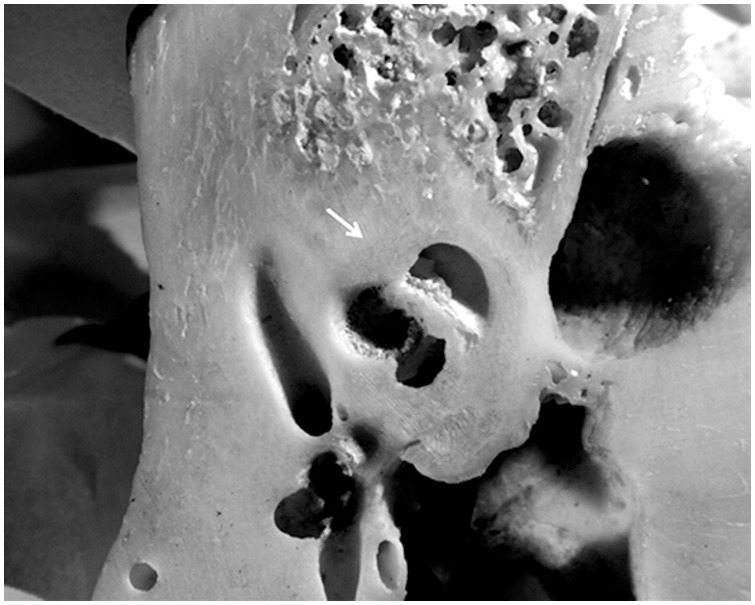
The otic capsule surrounding the vestibulo-cochlear organs of the inner ear is the one of the densest bone tissues in the human body and does not remodel after the age of 2. Here, the bone tissues in the inner ear are illustrated in a cross section of a petrous portion. The limit of the otic capsule is indicated by the arrow.

The petrous portion of the temporal bone (*pars petrosa*) has a formation process different than other compact bones of the human skeleton. The petrous portion is extremely robust and retains its morphology even after cremation or other intensive heat exposure, being one of the last bones of the body to burn [10]–[11] (Fig. 1). The otic capsule, surrounding the vestibulo-cochlear organs of the inner ear, is the one of the densest bone tissues in the human body, resembling enamel in strength (Fig. 2). Moreover, the high density and chemical strength makes it less exposed to contamination than other skeletal remains. The otic capsule is formed by endochondral ossification around the 16th–18th gestational week, and ossifies around the time of birth [12]. Hence, its fetal structure and chemistry are embedded in an unchanged primary form and do not remodel after the age of 2 [13]–[15]. This means that the Sr isotope ratios of the otic capsule may contain an archive of individual life history over the time of development (i.e. reflecting the diet of the mother during fetal stage and diet during the first 2 years of life) [16].

Here we present an intra-skeletal comparison of Sr isotope ratios in the otic capsule of the petrous portion and in dental enamel from premolars, formed around the age of 3–6 years [17]. We then apply the method to cremated human remains. This is the first study comparing Sr isotope ratios from cremated and non-cremated petrous portions as indicator for childhood origin. We aim to explore whether the Sr isotope ratio in the inner layer of the otic capsule in cremated petrous portions can be used as an indicator of diet consumed in fetal stage and the early years of life, and hence reflect childhood origin and human mobility. The petrous bone would thus be a proxy or supplement for dental enamel in cremated or burned human remains.

## Materials and Methods

The material used in the intra-skeletal comparative study of unburnt human remains consisted of bone tissue from the otic capsule of the petrous portion of the skull, and dental enamel from premolars sampled from 9 individuals (a total of 18 samples), all adults, both males and females with ages ranging from approximately 17 years to older seniles. The individuals were from early, middle and late medieval inhumation burials (1000–1536 AD) from the abandoned church cemetery, Tjærby Ødekirkegård, in the parish of Randers, Jutland, Denmark (K422–430; Table 1).

**Table 1:** Strontium isotope ratios (87Sr/86Sr) of petrous portions and dental enamel from Tjærby (non-cremated) and Rishøj (cremated) and of residues and leachates of petrous portions from crematin grave sites in the Fraugde region.

Sample no.	Site name	Burial form	Otic capsule	2 SD (ppm)	Dental enamel	2 SD(ppm)	Leachates	2 SD (ppm)	Grave	Sr concentration ppm
**KF 422**	KHM 899 Tjærby	Inhumation	0.71116	12	0.71026	25			A 1094, x303	117
**KF 423**	KHM 899 Tjærby	Inhumation	0.71085	29	0.71044	19			A 965, x239	53
**KF 424**	KHM 899 Tjærby	Inhumation	0.71069	30	0.71034	18			A 1240, x413	115
**KF 425**	KHM 899 Tjærby	Inhumation	0.71056	23	0.71088	15			A 1408, x697	212
**KF 426**	KHM 899 Tjærby	Inhumation	0.71028	25	0.71084	13			A 1623, x1015	92
**KF 427**	KHM 899 Tjærby	Inhumation	0.71043	22	0.70981	12			A 1426, x1858	162
**KF 428**	KHM 899 Tjærby	Inhumation	0.71065	16	0.71087	21			A 1400, x1859	121
**KF 429**	KHM 899 Tjærby	Inhumation	0.71150	21	0.71178	22			A 1342, x1892	34
**KF 430**	KHM 899 Tjærby	Inhumation	0.71012	20	0.71074	22			A 1486, x1948	136
**KF 442**	VSM 9048 Rishøj	Cremation	0.71159	6	0.71209	17	0.71310	31	Urn 2, x159	
**KF 431**	OBM 8441 ØB III	Cremation	0.71308	8			0.71068	8	QE	
**KF 432**	OBM 8414 KH II	Cremation	0.71020	9			0.71140	9	FX	
**KF 433**	OBM 8414 KH II	Cremation	0.70986	8			0.71016	8	HD	
**KF 434**	OBM 8414 KH II	Cremation	0.71812	8			0.71332	6	HF	
**KF 435**	OBM 8414 KH II	Cremation	0.71625	6			0.71148	13	JE	
**KF 436**	OBM 8414 KH II	Cremation	0.71087	6			0.71129	7	KK	
**KF 437**	OBM 8433 TB NV	Cremation	0.71032	6			0.70970	7	AHB	
**KF 438**	OBM 8441 ØB III	Cremation	0.71041	8			0.71220	6	PZ	
**KF 439**	OBM 8441 ØB III	Cremation	0.70937	9			0.70927	14	PS	
**KF 440**	OBM 8698 KG	Cremation	0.70929	5			0.70895	8	GJ	
**KF 441**	OBM 8441 ØB III	Cremation	0.71092	8			0.70989	8	PT	

The material used for testing the results on cremated human remains consisted of dental enamel from one burned 2nd molar and a burned petrous portion sampled from an adult male individual, buried in an urn grave at the site Rishøj near Viborg, Jutland, Denmark (K442/RH U2; Table 1). The material used for the case study, where only cremated portions of petrous bone were preserved, consisted of extracted bone tissue from the otic capsules of burned petrous portion from 11 individuals, 10 adults and one teenager, whereof only few could be sex determined. The individuals were from Late Bronze Age urn cremation burials (1100–500 BC) and early Iron Age cremation pits (500–1 BC) from 4 different grave sites in Fraugde parish, Funen, Denmark (K431–K441; Table 1).

The above skeletal materials are housed at the Laboratory of Biological Anthropology, The Institute of Forensic Medicine, University of Copenhagen, Denmark, on loan from the Museum of Viborg, Viborg, Denmark; Odense City Museums, Odense, Denmark; and the Cultural-historical Museum of Randers, Randers, Denmark, respectively.

### Sampling procedure

A sample from the otic capsule was taken from the 9 individual inhumed medieval skeletons, using the method described by Jørkov et al. in 2008 [16]: The petrous bone was drilled at a 90° angle into the otic capsule (0.5–0.8 cm down), between the internal acoustic meatus and the subarcuate fossa using a low speed (2-mm diameter) drill. Samples of dental enamel from the 9 inhumed skeletons and the single urn cremation burial were taken by removing the outer enamel surface with a diamond-coated micodrill. Small pieces of enamel were then sawn off with a 2 cm diamond-coated saw blade mounted on a hand-held microdrill. The clean enamel samples were rinsed with MilliQ (Millipore) water in an ultrasonic bath. The otic capsules from the 11 cremated individuals were separated from the cremated petrous portions using a 1 mm mechanical saw mounted on a (DREMEL model 300) drilling machine. This method was applied to prevent the burned and calcined bone to splinter, which occurs when drilling. Using the sawing method enabled sampling of the densest part of the central inner ear and resulted in fairly clean samples of intact otic capsules for the Sr isotope analyses.

### Sample treatment

The enamel samples were dissolved in a mixture of 1 ml 14N HNO3 and a few drops of H2O2 provided by Seasta in 7 ml Saville teflon beakers on a hotplate at 100°C over night. After evaporation to dryness, the samples were taken up by 3N HNO3 and added to a disposable pipette tip extraction column with a self-fitted frit and charged with 200 µl 100–150 mesh SrSpec (Eichrom/Trischem) resin. Sr extraction from these columns followed a slightly modified procedure of Horwitz et al. [18]. The petrous bone samples were leached with 1 M acetic acid during 10 minutes, and the residues were then attacked, dissolved and processed according to the same procedure as the enamel samples. The acetic acid leachates were dried separately and processed over the SrSpec ion chromatography.

Sr separates were loaded together with a T2O5-HFH3PO4-silicagel activator on rhenium filaments. Samples were analyzed in multi-dynamic mode on a VG Sector 54 IT thermal ionization mass spectrometer at temperatures between 1250 and 1350°C and signal intensities of 88Sr of between 1*10–11 to 4*10–11 A (corresponding to 1 to 4 Volts). Sr isotope measurements were normalized to 86Sr/88Sr = 0.1194 and corrected for the offset relative to the composition of the NBS 987 Sr standard, which we reproduce over a long time period at 87Sr/86Sr = 0.710238+/−0.000016 (2σ; n = 83). Procedure blank levels remained below 120 pg of Sr, an amount which insignificantly affects the Sr isotope compositions of the samples usually yielding Sr amounts in excess of several hundreds of nanograms.

## Results

We analyzed premolar enamel and bone tissue from the otic capsule from nine adult individuals from medieval inhumation burials from Tjærby near Randers, Jutland, Denmark (Table 1). Furthermore, the strontium concentrations of the otic capsule samples agreed well with the expected strontium concentrations found in bone tissues [19] indicative of non-diagenetic processes. We were also able to include one cremated individual from a Late Bronze Age urn cremation burial found near Viborg in Jutland, Denmark. The urn contained two well preserved maxillary molars with intact enamel and both petrous portions from an adult male (KF 442/RH U2), which is seldom found.

The ^87^Sr/^86^Sr ratios of premolar enamel range from 0.70981 to 0.71178 (average ^87^Sr/^86^Sr = 0.71066±0.0011) and the ^87^Sr/^86^Sr ratios of otic capsules range from 0.71012 to 0.71150 (average ^87^Sr/^86^Sr = 0.71067±0.000912). The second molar of the cremated individual (forms during the first 3–7 years of life, similar to premolar enamel) had an Sr isotope composition (^87^Sr/^86^Sr = 0.71209) in agreement with that of the otic capsule (^87^Sr/^86^Sr = 0.71159), confirming an expected low contamination of the bone tissue in the otic capsule and a good correlation of the values, even for cremated human remains (Table 1).

**Figure 3:**
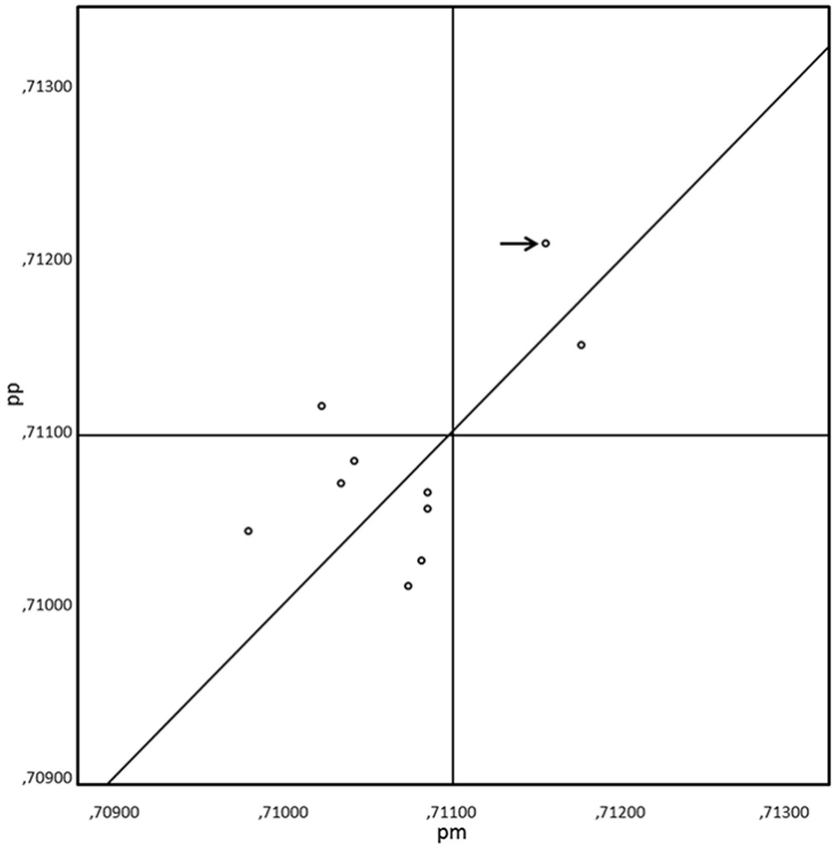
Bland and Altman plot showing the “line of equality”: the line on which the values would lie if Sr levels measured in premolar enamel (pm) exactly equaled the levels measured in the otic capsule of the petrous portion (pp). The values cluster around the line of equality, thus no systematic bias is indicated. The horizontal and vertical lines indicate the upper limit for strontium levels in Denmark (0.711; the lower level is 0.708). The two individuals in the upper right quadrant are thus non-Danish, while the individuals in the lower quadrant can be assumed to be Danish. The individual in the upper left quadrant could be either, depending on whether the enamel or the petrosal value is used. The cremated individual from Rishøjen (K442/RH U2) is indicated by an arrow.

**Figure 4:**
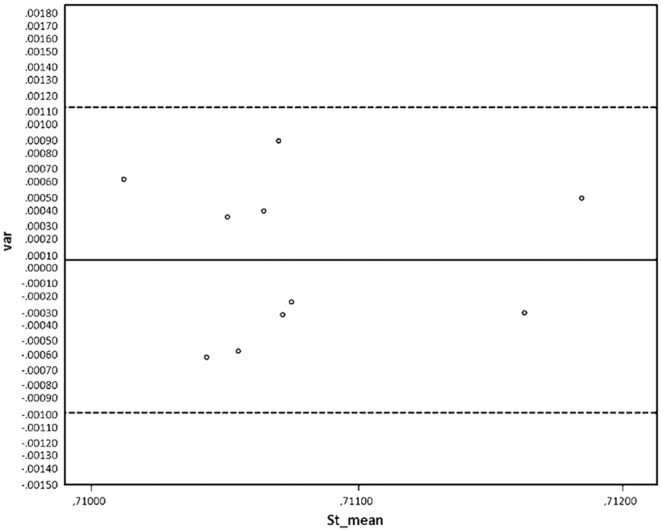
Bland and Altman plot showing the limits of agreement. The difference between the enamel and the petrosal values (var) are plotted against the mean value of the enamel and petrosal values (st mean). Again, no bias is seen. The mean difference is 0.00007 (indicated by solid horizontal line) and +/−2SD (+/−0.001076) is indicated by the horizontal dotted lines. No values lie outside these limits, indicating that measuring either the premolar enamel or the petrosal Sr isotopic ratios will give the other value within 0.001075 (2SD).

The pairs of Sr isotopic values of the individual’s premolar enamel and otic capsules are similar but not identical. The difference, valid for all the pairs, exceeds analytical precision and reproducibility (presently±0.00004) for Sr isotope analyses (figs. 3 & 4). There is, however, no systematic difference between the analysis of the otic capsule and premolar enamel of the respective individuals. The Intra Class Coefficient (ICC) is 0.594, p = 0.027 (F test). The best agreement is seen for the individual from grave A 1342, skeleton x 1892 (KF 429), with a Sr isotope ratio difference of only 0.00022 between the premolar enamel and the otic capsule. The highest divergence is for the individual from grave A 1094, skeleton x 303 (KF 422) with a Sr isotope ratio difference of 0.00090 (baseline values for the area around Tjærby, near the city of Randers, is of ^87^Sr/^86^Sr 0.708–0.709).

**Figure 5:**
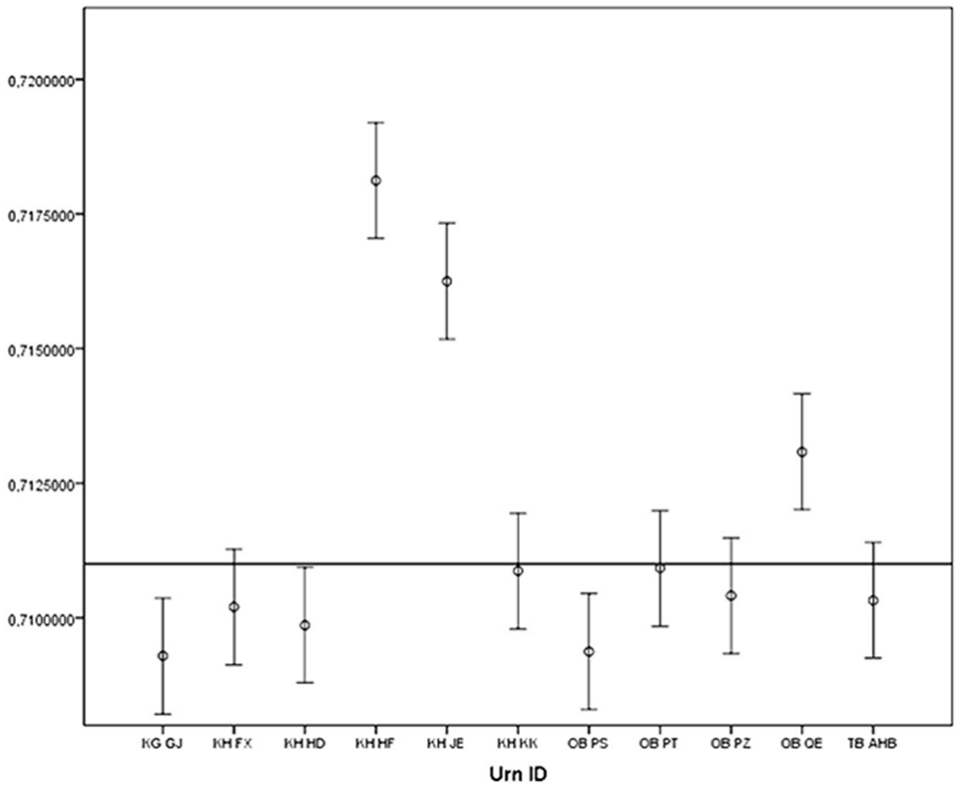
Plot of Sr isotope ratios for the cremated individuals. Error bars correspond to +/−2SD of the premolar enamel-petrous portion difference (0.001076) derived from the data in Figure 4. The individuals from the cremation graves KH HF, KH JE and OB QE can therefore be assumed to be non-local.

Based on the results from the 9 inhumed and the one cremated individual, and using the hereby established mean difference (including the 2SD range of the error), we then analyzed bone tissue from otic capsules of petrous portions from the cremated remains of 10 adults and one teenager (KH KK/KF 436), from four Late Bronze and Early Iron Age cremation grave sites near the village of Fraugde on Funen, Denmark. We have furthermore analyzed a water sample (creek) from the site of Fraugde in order to establish the local baseline. The water sample analyzed herein yielded a ^87^Sr/^86^Sr = 0.70939 (±0.00001), which is compatible with previous baseline investigations of the island of Funen where surface water samples yielded an average of ^87^Sr/^86^Sr = 0.70979 [20] and fauna samples define an average of ^87^Sr/^86^Sr = 0.70877 [21]. Leachates of all the cremated samples were further analyzed to monitor the removed Sr fraction (Table 1). Previous studies show that cremation result in an increase crystallite size [8] which may consequently decrease contamination processes in cremated bones in relation to non-cremated. The divergence in ranges of the non-local cremated individuals (K431, K434 and 435) indicates that these individuals came from different areas (Fig. 5).

## Discussion

That the Sr isotopic ratios from otic capsules and premolar enamel from the medieval inhumation burials are tightly correlated implies that Sr isotope analysis of the otic capsule can be used as a proxy for provenience applications. Some small divergence is due to the different sampling: sampling dental enamel can be done quite accurately, also in terms of the tooth formation times, whereas sampling the otic capsule bone will involve minute amounts of bone surrounding the otic capsule, and this bone may be formed later and may remodel as other skeletal tissue [1], [16]. Further small divergences between otic capsules and premolar enamel were expected because of a gradual shift in human diet during the first years of life from breast feeding to solid food. The differences likely therefore reflect that these two bone tissues form during two different stages of the early childhood (i.e. 3–6 years for premolar enamel, and fetal stage up to two years for the otic capsule). Nevertheless, the relatively low divergence must reflect that the buried individuals were sedentary during the first years of their lives. Moreover, the Sr concentrations in the otic capsules range from 35 to 213 ppm, which agrees with concentrations in skeletal and dental tissues reported for modern humans (50–300 ppm) [4].

We were thus able to determine non-local origins for three cremated individuals, although these individuals had no preserved dental enamel, as is the norm in cremations. Individual K431 was buried in an urn grave dated to 1100–900 BC. There is no archaeological indication of this individual being a non-local, but based on the Sr isotope ratios, the individual could originate from different areas, for example southern Sweden, the Danish island of Bornholm, or some areas in Germany and Poland [20]–[23]. K434 and K435 were buried in a cremation pit and an urn cremation pit, respectively, at a grave site dated to 700–500 BC, i.e. during the transition period between the Danish Bronze and Iron Ages. There is no archaeological indication of these individuals being non-locals in terms of grave goods and ceramics, but based on the Sr isotope ratios and the known cultural contacts in the period, an origin from Bornholm, southern Germany or Sweden is likely [20], [23], [24]. The individual from Viborg in Jutland, used for testing the method on cremated bone (K442/RH U2), was buried in an urn grave dated to 700–500 BC. There is no archaeological indication of this individual being a non-local, but based on cultural contacts in the period and the Sr isotope ratios, an origin from south of Denmark in the northern part of Germany is likely.

Future studies would involve more samples from different locales in order to test the method on a wider Sr range. Further, to fully explore any impacts of diagenesis, we would suggest testing petrosal bone and dental enamel for concentrations of other elements (e.g. Uranium), which does not occur naturally in bone and teeth, but do enter as diagenetic agents.
